# Numerical Investigations on Thermal Forming Limit Testing with Local Inductive Heating for Hot Forming of AA7075

**DOI:** 10.3390/ma14081882

**Published:** 2021-04-09

**Authors:** Franz Reuther, Thomas Lieber, Jürgen Heidrich, Verena Kräusel

**Affiliations:** Fraunhofer Institute for Machine Tools and Forming Technology IWU, 09126 Chemnitz, Germany; thomas.lieber@iwu.fraunhofer.de (T.L.); juergen.heidrich@iwu.fraunhofer.de (J.H.); verena.kraeusel@iwu.fraunhofer.de (V.K.)

**Keywords:** formability prediction, hot forming, AA7075, TFLD, finite element analysis, cruciform biaxial tensile tests

## Abstract

Forming 7000-series aluminum alloys under elevated temperatures is particularly attractive due to their increased formability. To enable process design by finite element simulation for hot forming, strain-based criteria, such as temperature-dependent forming limit diagrams (TFLD), can be consulted to assess forming feasibility. This work numerically investigates the extent to which in-plane experimental concepts with partial inductive heating are suitable for detecting discrete failure points in TFLD. In particular, an alternative to the currently widely used thickness-reduced specimen geometries was created for cruciform specimens under biaxial tension. First, the temperature-dependent and strain-rate-dependent flow behavior was investigated for AA7075 under uniaxial tension. A heat source model for partial inductive heating was inversely parameterized based on heating experiments. Subsequently, the test procedures were simulated with different specimen geometries under discrete strain conditions. Different concepts were discussed for deriving a suitable specimen shape for the biaxial tension case, and the influence of different notch and slot forms were shown. The simulations showed that partial inductive heating was suitable to induce failure situations, thus creating TFLDs. For the biaxial tension case, a sufficiently large temperature gradient was required to use cruciform specimens without thickness reduction.

## 1. Introduction

High-strength aluminum alloys of the 6000 and 7000 series have increasingly raised the interest of the automotive industry due to their high strength to weight ratio and stiffness to weight ratio, improved corrosion resistance, joinability and recyclability [1]. However, since the forming capacity is limited for the higher-strength aluminum alloys of the 7000 series at room temperature, various manufacturing technologies have recently been developed for processing at warm or hot forming temperatures [2]. In particular, the combination of forming and quenching processes is considered here, in which a solution-annealed blank is simultaneously formed in a cooled die and quenched. Compared to forming at room temperature under W-temper conditions, the higher cooling rates during the forming process result in increased ductility and significantly higher dimensional stability, as well as higher component strength after artificial aging [3]. Virtual methods are state of the art for an industrial method development for planning, design and optimization of such hot forming processes. In recent years, numerous studies on the modeling of thermomechanical problems in finite element simulation have contributed to a considerable increase in knowledge [4,5]. These processes can generally be well computed numerically by systematically modeling heat transfer mechanisms along the process chain, temperature and strain-rate-dependent material models, and by considering tribological conditions. Approaches based on forming limit estimation also already exist for the prediction of forming process limits. Forming Limit Curves (FLCs) can be used to estimate the tolerable forming degree under varying strain states. This approach is widely used in industrial applications for estimating the forming feasibility, especially in the field of conventional sheet metal forming at room temperature.

The standardized tool-bound Nakajima or Marciniak tests according to DIN EN ISO 12004 [6] are generally used for detecting forming limit curves at **room temperature**. In these tests, specific forming states are imposed on the sheet metal semifinished product until they reach the limit of the respective tolerable forming change by reaching an instability state (necking or crack). The measurement of the locally present degree of forming is usually carried out using optical strain measurements [7]. This established approach’s essential process-specific limitations lie in the restriction to linear strain paths [8]. Furthermore, the shape of the FLC is decisively influenced by sheet thickness, rolling direction, friction, punch speed and the forming history [9]. The choice of test method influences the shape of the FLC due to the different tooling concepts and specimen shapes that result in different load conditions on the material [10]. According to Raghavan [11], a fundamental distinction can be made between in-plane methods (tensile test, plane-strain tests, plane torsion tests, shear tests, Marciniak cup tests) and out-of-plane procedures (hydraulic bulge test, Nakajima test, elliptical bulge tests), which can be used to detect discrete points on the FLC. A detailed comparison of the different experimental concepts can be found in [8].

The determination of forming limit curves at **elevated temperatures** is currently not standardized. Numerous investigations are known in which the recording of temperature-dependent forming limit curves (TFLC) was carried out through Nakajima or Marciniak tests. Naka et al. [12] and Turetta et al. [13] used Marciniak and Nakajima tests, respectively, with heated punches acting from above, and measurement systems for strain and temperature located below the specimen. The sample was heated to temperatures of up to 300 °C [12] via stamp contact or through inductive heating [13]. Lechler [14] also used a Nakajima tool for testing manganese-boron steels with which the FLC could be determined up to 650 °C, whereby the punch, die, and blank holder could be separately heated using heating cartridges. The austenitization of the blanks was carried out upstream in a chamber furnace. In this context, particular attention was drawn to the problem of off-center specimen failure, which is a consequence of the more significant frictional influences under higher temperatures. Ensuring a homogeneous temperature distribution in the blank’s measuring area is also challenging with such tool-based test concepts. Continuous convection and radiation losses, and the different contact points between the blank and heated tool components, can cause inhomogeneous temperature distributions. Similar test strategies are known for testing aluminum alloys of the 7000 series to detect TFLD by out-of-plane tests [15]. Xiao et al. [16] and Ying et al. [17] also reported on a necking behavior outside the specimen center and the immense influence of friction using the example of AA7075. In this context, large differences occurred in TFLD results for AA7075, which according to Ying et al. [17] were primarily due to different test conditions, semifinished products, and thermal process routes.

For these reasons, a **frictionless identification of the TFLD** is a reasonable alternative to conventional tool-bound out-of-plane testing methods, as presented for room temperature in [18,19], among others. At elevated temperatures, biaxial tensile tests are successfully used for yield locus determination, as shown, for example, by Merklein et al. [20] for a magnesium alloy AZ31 up to 310 °C and Naka et al. [21] for aluminum alloy AA5083 up to 300 °C. Numerous specimen shapes also exist for yield locus determination at room temperature in biaxial tensile tests [22,23]. Other approaches were developed to determine FLC points in the biaxial tension test, such as the sample shapes proposed by Zidane et al. [18] or by Leotoing and Guines [24], which are primarily related to room temperature studies. By partially reducing the specimen in the thickness direction, a necking and failure situation can be successfully shifted toward the specimen’s center while avoiding premature specimen failure at the notched areas. The detailed studies of Zidane et al. also show that appropriate sample shape optimizations can detect a wide range of different strain states. However, this approach’s disadvantages lie in the relatively costly specimen fabrication and in the limitation of the sheet thicknesses that can be considered in association with the thickness reduced specimen geometry. A transfer of this basic approach to thin sheet semifinished products was investigated by Jocham et al. [25] by joining several layers of thin sheet specimens through adhesive bonding. Discrete FLC points were successfully detected through simulations and experiments using various specimen shapes with different edge fillets, slot positions and thickness reductions. Shao et al. [26,27] investigated a tempered experimental design for forming limit testing with biaxial tensile specimens. Using AA6082 as an example, forming limit tests have already been successfully carried out in this context at a temperature range of 370–510 °C with conductive sample heating. Again, using cruciform specimens with a thickness reduction was necessary to induce a failure situation in the specimen center. According to the current state of the art, there are no publications on thermal forming limit testing of cruciform specimens without thickness reduction. Furthermore, exclusive local heating of the specimen center has not yet been investigated in this context.

## 2. Objective

**Figure 1:**
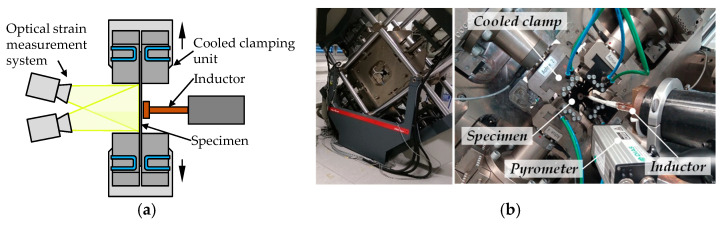
Frictionless formability testing at elevated temperatures: (**a**) test principle with local inductive specimen heating and optical strain measurement in cross-sectional view; (**b**) biaxial tensile testing machine and experimental setup.

The aim of this numerical study is the investigation of forming limit testing for hot forming of higher strength aluminum alloys by specimens without thickness reduction only heated by local induction. In order to overcome the mentioned disadvantages of the existing out-of-plane test concepts, forming limit testing was also conducted by **frictionless in-plane** uniaxial or biaxial tensile tests in a biaxial tensile testing machine (Figure 1b). However, this new concept, which is contrary to the state-of-the-art methods, comprises **specimens without central reduction of thickness** combined with only **local specimen heating by induction**. This method reduces the effort for specimen preparation and avoids an influence of the formability due to thickness reduction. This paper numerically investigates which sample shapes, combined with local inductive heating, are suitable to determine discrete points of TFLD (Figure 1a).

For this purpose, the basic thermal process routes are discussed first. Subsequently, the material flow properties are characterized by tempered tensile tests. The derived flow curves for discrete testing temperatures and strain rates form the basis for modeling the material behavior in forming simulation. In order to model the process holistically, an analytical heat source model is also parameterized to represent local inductive heating. Then, different specimen shapes are evaluated and compared in the simulation of the testing process. The result is the recommendation of sample geometries for the detection of TFLD under discrete forming conditions, including partial inductive sample heating.

## 3. Materials and Methods

### 3.1. Preliminary Considerations for Thermal Process Routes

**Figure 2:**
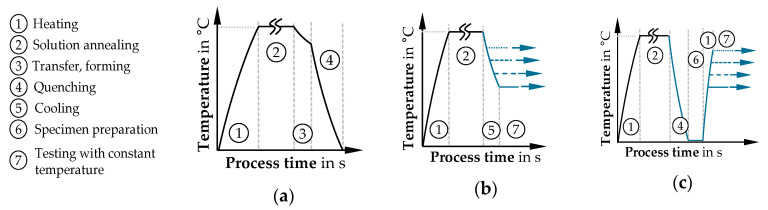
Thermal process routes for hot forming of 7000-series aluminum: (**a**) typical thermal process route for hot forming; (**b**) isothermal material testing immediately after solution annealing; (**c**) isothermal material testing after solution annealing, quenching and heating up to target temperature.

The higher-strength aluminum alloy AA7075 in the initial T6 condition was selected as the material to be investigated with a sheet thickness of 2.0 mm (supplier: Hydro Aluminium Rolled Products GmbH, Hamburg, Germany). First, the thermal process route must be discussed to guarantee process-relevant conditions. According to Figure 2a, the target process is hot forming [28]. Compared to forming at room temperature, significantly improved forming properties result from the process combination of heat treatment and forming under quenching conditions, first presented by Garrett et al. [29], also known as the hot form quench (HFQ) process [30]. The process first requires a solution annealing step (AA7075 T6: 480 °C at 15 min [31]), followed by transfer of the heated blank to a cooled tool, where simultaneous forming and quenching is performed. According to Degner [3], the relevant temperature range for forming is 400–100 °C. A critical cooling rate must be achieved during quenching to suppress premature precipitation formation in favor of a supersaturated state [3]. The required cooling rate in the range from 400 to 290 °C is 100 K/s for AA7075 T6 [31]. The tool opening and the following component removal takes place after cooling to below the artificial ageing temperature to avoid hardening effects [32]. The industrial production chain is completed by subsequent artificial ageing to achieve the required component strength [33].

Material testing for this forming process is usually also realized by an initial solution annealing process, followed by cooling of the specimen to the desired test temperature before the isothermal material test starts, e.g., in the uniaxial tensile test (Figure 2b). In numerous cases, only a temperature range above 300 °C can be tested to avoid precipitation formation due to excessively low cooling rates. From an experimental point of view, the exact replication of the characteristic thermal process route is challenging; in particular ensuring a sufficiently high cooling rate down to the test temperature.

Due to these problems, an alternative thermal process route C is discussed (Figure 2c), which is regarded as a compromise solution, especially in the context of this testing aim with local inductive heating. According to Figure 2c, external solution annealing is performed followed by a quenching process in a cooled plate-tool to first set a W-temper condition in the samples holistically. After specimen preparation for optical strain measurement and clamping, only the specimen center is inductively heated to the target temperature, followed by testing under isothermal conditions. The rapid heating and short sample preparation time minimize diffusion-controlled precipitation processes. Nevertheless, an influence by incipient precipitate formation (*η*, *η*′ precipitates [34]) can be assumed after this thermal process route.

### 3.2. High-Speed Tensile Testing and Constitutive Material Modeling

**Figure 3:**
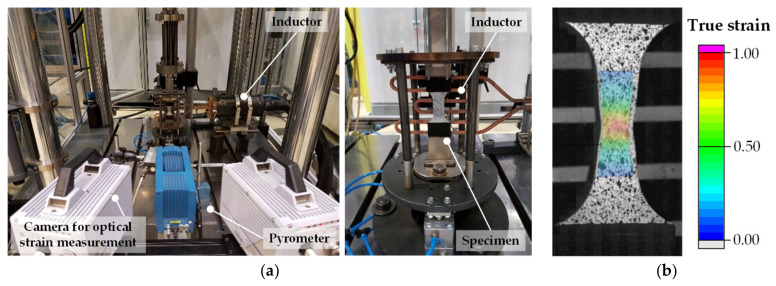
High-speed tensile tests for discrete temperatures and strain rates: (**a**) experimental setup with local inductive heating of the specimen area; (**b**) optical strain evaluation of an exemplary tensile test at 400 °C and 1.0 s^−1^.

High-speed tensile tests were performed on a Zwick Roell HTM 16020 (ZwickRoell GmbH & Co. KG, Ulm, Germany) according to the test setup in Figure 3a. Thermal process route C (Figure 2c) was selected to ensure boundary conditions identical to the subsequent simulations of forming limit testing. The tensile test specimens with a width of 16 mm were prepared by electrical discharge machining and solution annealed externally in a chamber furnace at 480 °C for 5 min. Quenching in a cooled plate-tool ensures a cooling rate of 100 K/s to produce a homogeneous W-temper condition. A stochastic pattern for strain evaluation (Figure 3b) was applied within 20 min after quenching. Inductive sample heating to target temperatures (200, 280, 360, 400 and 450 °C) was performed at an average heating rate of 10 K/s. Then tensile tests were performed under discrete strain rates of 0.1, 1.0 and 5.0 s^−1^ after a holding time of 30 s to homogenize the temperature field.

The experimentally determined data points for a strain rate of 0.1 s^−1^ were approximated for each test temperature using Equation (1).

(1) $$\sigma =A-(A-B)e^{-C\cdot \varepsilon_{pl}{}^{D}}$$

In addition, the strain rate dependence was modeled by scaling this yield stress according to Equation (2) via the strain rate sensitivity *m* and the reference strain rate $\dot{\varphi }$_*ref*_.

(2) $$\sigma_{\mathrm{flow}}=\sigma {\left(\dot{\varphi }/{\dot{\varphi }}_{ref}\right)}^{m}$$

**Figure 4:**
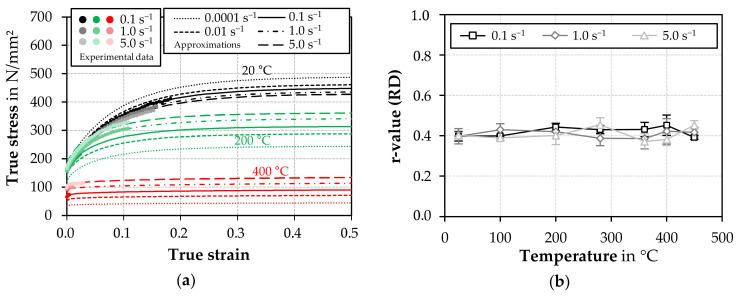
Results of the tensile test at different temperatures and strain rates: (**a**) experimental flow curve data and flow curve approximations for selected test temperatures; (**b**) r-values for different test temperatures and strain rates in rolling direction (RD).

The experimentally determined data points and the derived yield curve approximations serve as examples for 20 (W-temper), 200 and 400 °C in Figure 4a. Table 1 lists the coefficients of the flow curve approximations and strain rate sensitive modeling.

A negative strain rate sensitivity was observed at 20 °C due to the portevin-le-chatelier effect [35]. With increasing temperature, the strain hardening capacity decreases and the strain rate sensitivity increases continuously. Above 280 °C, nearly steady flow curves were identified by dynamic recovery. Besides, r-values dependent on temperature and strain rate in the rolling direction (RD) were also determined from the evaluations of the strain across the width (Figure 4b). The plastic anisotropy behaves nearly constant over the measured temperature range and independent of the strain rate.

Additional r-values and initial yield stresses diagonal and transverse to the rolling direction were determined from tensile tests at 0.1 s^−1^ at room temperature (W-Temper) to parameterize an anisotropic yield locus model. For this purpose, the Barlat2000 [36] yield locus model was used for adequate modeling of the yield stress and r-value anisotropy (Table 2). Isotropic behavior was assumed for the flow properties under biaxial stress conditions.

### 3.3. Pre-Estimation of the Temperature-Dependent Formability

**Figure 5:**
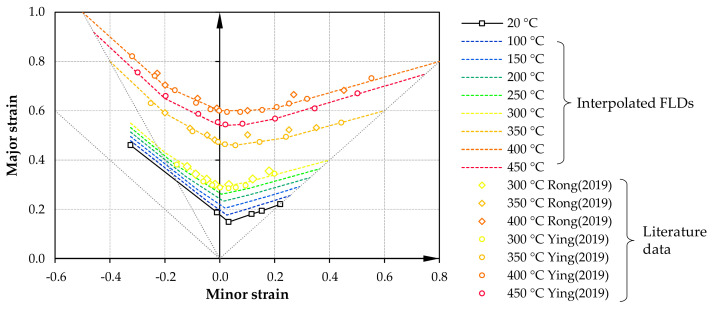
Assumption for TFLDs for AA7075 according to Rong et al. [37] and Ying et al. [17].

A preliminary estimation of the formability was required within the simulations for simulating different sample concepts. An assumption of formability was derived according to Figure 5 based on previously published work for AA7075 by Rong et al. [37] and Ying et al. [17]. Further TFLDs were interpolated up to 450 °C based on an experimentally measured FLC for room temperature. The formability increases only comparatively slightly up to a temperature of 300 °C. Up to a temperature of 400 °C, a significant jump in the TFLD can be seen before it tends to decrease slightly again to above 400 °C.

### 3.4. Approaches and Boundary Conditions of Finite Element Model

Utilizing the simulation software LS-DYNA (Release R12, LSTC, Livermore, CA, USA), a shell-based model for the testing process was built using the approaches and boundary conditions from Table 3. The kinematics of the specimen clamping areas was realized by a translation boundary condition, through which the clamping jaws were also moved at an initially constant speed. Contact modeling was relevant here only for reasons of thermal boundary conditions to account for the continuous heat flow towards the cooled jaw regions. The thermal expansion due to the heating process was not initially considered here within the scope of the model. The specimen was also segmented radially into several individual model parts to assign locally different FLCs according to Figure 5, corresponding to the mean temperature of the segment. This approach is necessary since there is currently no direct method implemented in LS-DYNA to account for temperature dependent FLCs.

**Figure 6:**
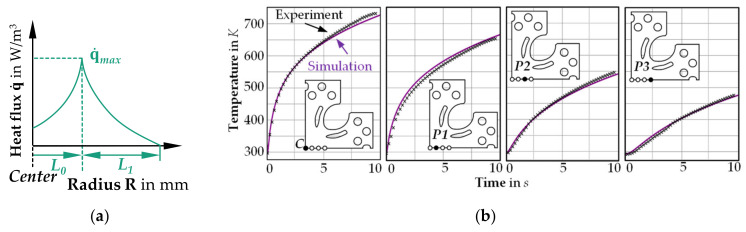
Analytical modeling of the heat source: (**a**) approach for modeling the location-dependent heat flow as a function of the radius; (**b**) comparison of the experimental and simulated temperature-time curves at different evaluation points, using the inversely identified parameters of the heat source model.

In order to reproduce the heat distribution in the specimen by local inductive heating, an analytical heat source approach was chosen in LS-DYNA. For calibrating this model, local inductive heating tests with cruciform specimens were carried out to measure temperature-time curves at different evaluation points. Subsequently, various heat source approaches were investigated regarding their accuracy, of which the one shown in Figure 6a proved promising. According to Equation (3), a location-dependent heat flux was defined whose maximum value ${\dot{q}}_{max}$ became effective at a distance *L*_0_ from the sample center. *L*_1_ describes the maximum radial effective zone of the heat source to the outside:

(3) $$\dot{q}=f(R)={\dot{q}}_{max}\times \left[1-{\left(\frac{L_{0}-R}{L_{1}}\right)}^{M}\right],$$

The inverse determination of the coefficients from Equation (3) was performed by recalculating the experimental induction tests utilizing the software LS-OPT V6 (LSTC, Livermore, CA, USA) (Figure 6b). The experimental results of the induction tests were very well reproduced with the identified parameters from Table 4.

### 3.5. Simulation Stages of the Testing Procedure

The test process for frictionless detection of TFLDs is designed according to thermal process route C (Figure 2c). This design assumes an externally completely solution-annealed and quenched specimen. Therefore, the stepwise simulation of the entire test process was divided into the individual stages of heating to the test temperature, holding the temperature, and testing the specimen at a constant center temperature. Since the actual heating step is completed within a few seconds, the holding time before the actual start of the test required realistic estimation. An equilibrium state is only gradually established due to the continuous heat supply in the specimen center and the heat conduction processes towards the cooled clamping jaws. Here, a holding stage of 30 s was assumed. For the simulation-based design and evaluation of different specimen shapes, test trials at 200 and 400 °C are considered as limit cases in the following.

## 4. Results and Discussion

### 4.1. Specimen Geometries for Uniaxial Tension and Plane Strain Testing

**Figure 7:**
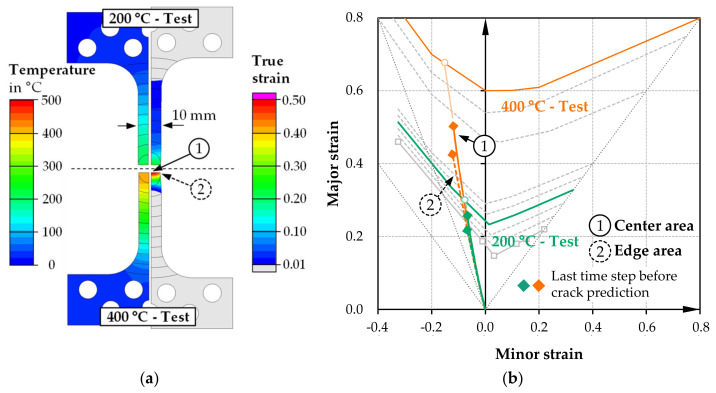
Simulation results or uniaxial tensile testing specimen for 200 and 400 °C tests: (**a**) temperature distribution at start of loading, plastic strain distribution immediately before center crack; (**b**) forming limit diagram with strain paths of center and edge area.

**Figure 8:**
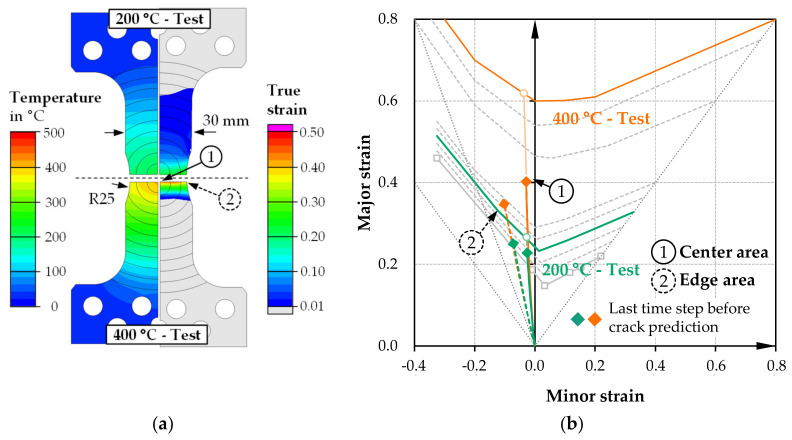
Simulation results of plane strain testing specimen for 200 and 400 °C tests: (**a**) temperature distribution at start of loading, plastic strain distribution immediately before center crack; (**b**) forming limit diagram with strain paths of center and edge area.

The comparatively easily attainable strain states of uniaxial tension and plane strain were selected for the simulation setup’s initial testing. The corresponding specimen shapes are shown in Figure 7; Figure 8. In the first case of the uniaxial tensile specimen, inductive heating via the parameterized heat source model is already completed after 0.5 s (200 °C), respectively 1.3 s (400 °C). After 30 s of holding at this respective temperature level in the specimen center, a quasistatic state is set due to heat conduction processes, convection or radiation losses and the heat transfer into the clamping jaws. Figure 7 shows the resulting temperature gradient immediately before the load application for the two tests (200 and 400 °C). In the actual testing process, the clamping areas constant speed subsequently causes elastic-plastic deformation of the notched specimen area.

Due to the larger temperature gradient in the 400 °C test, only a relatively small area plasticizes immediately around the inductively heated zone. According to the TFLD criterion (Figure 5), cracking is initially predicted in the center in the two cases. The major strain-minor strain diagram shows the strain distribution for two characteristic areas in the last time step before crack prediction. In the two temperature cases, a typical strain state for uniaxial tension with negative minor strains is present in the specimen center and in the plasticized zone’s edge region. Local inductive heating successfully supports plastic strain concentration in the specimen center.

The plane strain geometry in Figure 8 also shows a different deformation behavior of the notched area caused by the temperature gradient of the 200 and 400 °C tests. In both cases, the specimen center shows good plane-strain behavior, while in edge regions with negative minor strains, behavior equivalent to uniaxial tension tends to result due to the width constriction in these areas. The crack prediction according to the TFLD criterion results again in the center in both cases. Furthermore, this failure situation resulted from the notched specimen shape but, simultaneously, the local inductive heating supports the concentration of the deformation in the specimen’s center. Since the inductively heated area is now smaller than the specimen width, temperature gradients towards the edge are also visible in the relevant forming area for the first time. In the edge area, the temperature at the start of loading (specimen center 400 and 200 °C) is 29 °C, respectively 24 °C lower, which also reduces the formability towards the edge. In the case of the plane-strain specimens, there have been no impairments in the form of initial edge cracking.

### 4.2. Specimen Geometries for Biaxial Tension

**Figure 9:**
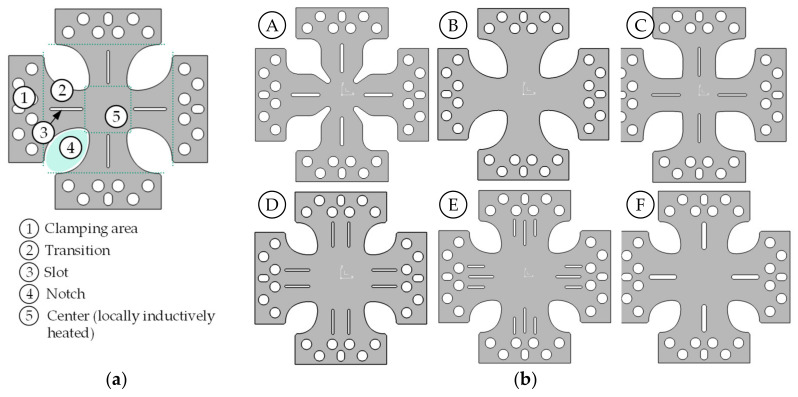
Types of different biaxial specimen geometries: (**a**) basic sample shape including naming of the characteristic geometric components; (**b**) different characteristic sample shapes.

The clearly challenging design of a specimen geometry for the biaxial tension case is not only subject to the temperature-dependent flow behavior of the material, but also to a strong temperature dependence of the formability. Conventional test concepts for biaxial tensile specimens without partial thickness reduction show, in testing at room temperature, that notched specimen areas or areas around inserted slots first plasticize before a deformation can be recorded downstream in the specimen center. These notched regions generally tend toward plane strain behavior. Thus, they are significantly more likely to reach a failure-critical strain level than the biaxially drawn specimen regions of the center. To counteract this problem, the current state of research and development often employs specimen geometries with partial thickness reduction, which induce a failure scenario at different temperatures in the specimen’s center [40,41]. However, the thickness reduction represents an intervention in the surface structure of the semifinished product, which may certainly have an influence on the formability. For this reason, an approach was chosen as an alternative to adjust temperature grading by means of local inductive heating in such a way that, because of the resulting property grading, an isolated failure situation could also be provoked in the specimen center. In this context, the simulation comprised testing of numerous sample geometries with different notch sizes, notch radii and slots in the transitions (Figure 9).

In principle, a categorical distinction has to be made between the behavior at 200 and 400 °C when interpreting the results. The 400 °C variant implies a relatively large temperature gradient so that the flow properties (yield stress level and strain hardening behavior) differ significantly between the inductively heated zone and the surrounding areas (slot, notch tip, and transition areas). For many specimen shape concepts, it is evident, even at low loading levels, that this temperature distribution causes the specimen center to plasticize quickly and the plastic strain to increase continuously. The shape and size of the notch only have limited influence on the deformation behavior, but they significantly affect the required force. Additional inserted slots can relieve the notched areas and homogenize the strain field in the specimen center. In the case of inductive heating of the specimen center to only 200 °C, the temperature gradient between specimen center and edge regions is reduced, resulting in decreased differences in yield stress level and strain hardening behavior. The specimen center is only slightly softened compared to the edge regions, and the concentration of plastic strain observed at 400 °C in the specimen center does not occur here. Similar to the case of plane-strain specimens and uniaxial tension specimens, this condition causes the specimen to be plastically deformed over a much larger area, which can lead to localization and cracking, especially in the transition areas. The average temperature in the smallest cross-section of the transition is usually only 40–50 °C lower than in the specimen center (200 °C). Additional introduced slots weaken the cross-section of the transition beyond this point so that here the transitions usually break off and the specimen center is hardly plastically deformed. The notch shape can have a positive influence if the smallest cross-section of the transition is located as far away as possible from the center of the specimen, which implies a greater temperature gradient. This effect can be implemented, for example, in the form of drop-shaped notch forms (Figure 9 specimen forms B–F). Notch shapes with a circular tip (specimen shape A) are unfavorable for this reason since this cross-section tends to be closer to the specimen center in this case.

**Figure 10:**
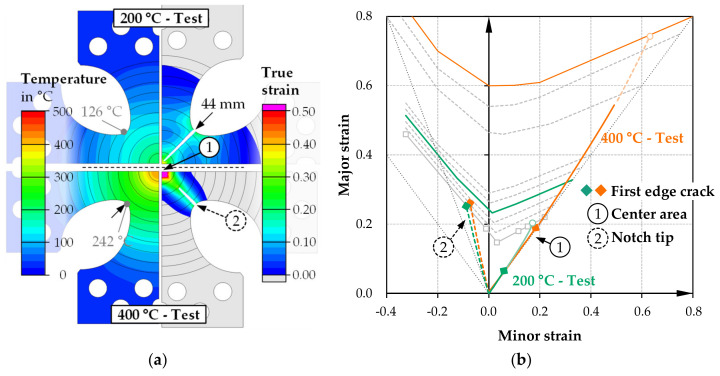
Simulation results of biaxial testing specimen B for 200 °C and 400 °C test: (**a**) temperature distribution at start of loading, plastic strain distribution immediately before center crack; (**b**) forming limit diagram with strain paths of center and notch tip area.

Figure 10 shows the detailed simulation results for the sample shape B. The temperature from the specimen center to the notch tip is reduced to 126 °C (200 °C test), or 242 °C (400 °C test) at the start of loading when using this comparatively simple drop-notched specimen without additional slots. In the 200 °C test, a large part of the transition plasticizes with a strain maximum in the notch tip area. When this point reaches the temperature-dependent forming limit level (ε_v_ ~ 23%), cracking starts. At this point, the true strain in the specimen center is just ε_v_ = 12%. During the progression of the crack, which continuously advances diagonally into the specimen center, the true strain in the specimen center continues to increase, but the level of the TFLD for 200 °C is not reached separately from the outer cracks.

The incipient crack in the notch tip occurs much later (ε_v_ ~ 27%) in the test at 400 °C inductive specimen heating. On the one hand, the specimen deformation is proportionally more strongly implemented via a deformation of the specimen center (ε_v_ ~ 36%). On the other hand, the forming limit increases due to the higher temperature in the notch tip. In this case, the crack also runs diagonally toward the center with continuously advancing elongation of the specimen center. However, the center reaches the forming limit for 400 °C separately before the crack completely penetrates the specimen’s center. The idealized simulation model naturally implies uniform crack initiation due to the assumed symmetry, which would have to be tested experimentally first. Despite the considered anisotropy of the material model, the advancing crack formation does not negatively affect the specimen center’s strain behavior so that a largely linear strain path is predicted. Under these conditions, it would be possible to determine the temperature-dependent forming limit in the experiment despite external cracking.

Other specimen shapes were investigated to prevent or reduce cracking, resulting in various slot arrangements, which were particularly suitable for retarding or suppressing cracking in the notch tip area. With a center slot extending further into the specimen center (sample shape E), the cross-section of the specimen center can be reduced so that the break-off of the transitions is initially avoided at medium test temperatures. This method also allows relief of the notch tip area, which is also conducive for implementing higher plastic strain in the specimen center. It is required that the slots must not extend too far into the tempered region but reduce the specimen cross-section of the center only insofar that the plastic deformation in the transitions does not reach the forming limit level. Otherwise, cracking would be initiated relatively quickly at the slot tips due to the increasingly lower temperature gradient towards the specimen center, and the plastic strain in the specimen center remains low. In the case of the 200 °C variant, no specimen shape was detected for various slot lengths and arrangements that fulfilled all the necessary boundary conditions in terms of deformation and failure behavior. Avoiding transition tearing off and relieving the notch tip area can be carried out, but even then, no isolated failure of the specimen center can be realized. In summary, testing at 200 °C implies too low a temperature, which also implies a low gradient of flow properties, making it impossible to achieve the objective with the testing strategy discussed here.

**Figure 11:**
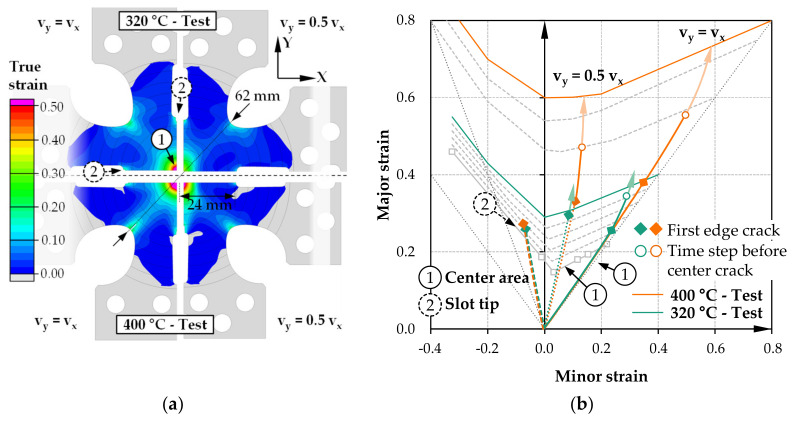
Simulation results of biaxial testing specimen F for 400 and 320 °C test: (**a**) plastic strain distribution immediately before center crack for two loading speed conditions; (**b**) forming limit diagram with strain paths of center and slot tip area.

As the best compromise for a temperature-variable test specimen shape F is derived. Figure 11 shows the results of the strain distribution and the forming limit diagram. The relatively wide central slot (4 mm) protrudes into the specimen center just enough to ensure that neither the transitions tear off nor the notch tip area tears excessively in a temperature range of 300–400 °C.

Figure 11 illustrates the results for a 320 and a 400 °C test, each as an ideal biaxial tension case (v_y_ = v_x_) and with reduced loading speed in the y-direction (v_y_ = 0.5 v_x_), to also verify the specimen suitability for a deformation state between plane-strain and biaxial tension. Both the notch tip area and the slot tip area are plastically elongated under advancing loading, with a simultaneous increase in elongation at the specimen center. In all cases, crack initiation is predicted in the slot tip area. For the ideal biaxial case, the plastic strain at this point in the specimen center is ε_v_ = 48% (320 °C) or ε_v_ = 72% (400 °C). With subsequent widening of the crack region, the plastic strain in the specimen center increases until reaching isolated central specimen failure. The outer cracks in the area of the slot tips penetrate the specimen center only slightly. This behavior is also confirmed for the second case of reduced loading speed in the y-direction. In some cases, crack initiation in the slot tip is even completely avoided (320 °C case), and completely isolated failure of the specimen center occurs. Based on the simulation studies, specimen shape F can be recommended for a test range of 300–400 °C. The investigated cases predict isolated specimen failure under local inductive heating, demonstrating the possibility of detecting temperature-dependent points of the FLC in the range from plane-strain to biaxial tension with cruciform specimens without thickness reduction.

## 5. Conclusions

This work numerically investigated the extent to which in-plane experimental concepts with partial inductive heating are suitable for forming limit testing for hot forming of AA7075. In summary, the following results were obtained:

1. Local inductive heating could be calculated in the simulation with sufficient accuracy via an analytical heat source model.
2. Forming limit testing under uniaxial tension and plane-strain conditions was ensured with suitable specimen shapes throughout the investigated temperature range from 200 to 400 °C. For the two strain states, isolated central crack initiation was predicted in the simulation.
3. For testing cruciform specimens without thickness reduction under biaxial tension, a sufficiently large temperature gradient between specimen center and transition must be ensured to induce an isolated failure situation of the specimen center.
4. The simulation studies were used to derive a promising cruciform specimen shape with slots for a test range of 300–400 °C, which allowed isolated specimen failure in the center while avoiding external cracking.
5. The slotted cruciform specimen was also suitable for determining discrete points on the temperature-dependent FLD for the range of plane-strain conditions up to the biaxial tension point.
6. In summary, the simulation was successful in testing the new concept of locally inductive heated specimens without thickness reduction for in-plane forming limit testing of AA7075.

Based on this simulation study, future work must provide experimental evidence of the deformation and failure behavior of the specimens. The real deformation behavior can only be modeled approximately due to the influences of discretization or symmetry assumption and the limits of the TFLD criterion used here. A possible influence of the thermal expansion must also be checked experimentally. Finally, future investigations shall comprise the transfer of the experimental methodology to a more realistic thermal process route with solution annealing directly in the clamping situation.

## Figures and Tables

**Table 1:** Identified values of the flow curve approximation and the strain rate sensitive model.

Temperature (°C)	A (N/mm^2^)	B (N/mm^2^)	C	D	m	${\dot{\varphi }}_{0}$ (s^−1^)
20	449.8	161.9	9.536	0.929	−0.0121 ^1^	0.1
200	314.2	155.8	8.622	0.769	0.0364	0.1
280	184.9	110.1	5.502	0.611	0.0450	0.1
360	103.3	85.9	3.449	0.447	0.0624	0.1
400	89.0	65.3	2.583	0.351	0.1034	0.1
450	63.0	41.9	1.721	0.314	0.1869	0.1

^1^ The strain rate sensitivity m was assumed 0.001 for 20 °C in the simulation since negative values would lead to diffuse local deformation of individual mesh regions when they change from the elastic to the plastic state.

**Table 2:** Measured and assumed parameter values for Barlat2000 yield function.

Parameter	σ_45_/σ_0_	σ_90_/σ_0_	r_0_	r_45_	r_9__0_	σ_b_/σ_0_	r_b_	M
Unit	—	—	—	—	—	—	—	—
Value	0.953	1.014	0.412	0.780	0.450	1.0	1.0	8

**Table 3:** Main simulation parameters and approaches.

**Process Parameters**	**Value/Approach**	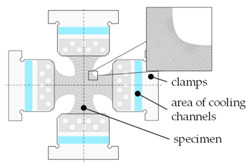
Constant speed of the clamps	0.033 mm/s	
Maximum force per loading axis	30 kN	
Sheet thickness	2.0 mm	
**Specimen Modeling**		
Element side length	0.5 mm; 0.25 mm only in notched areas	
Shell formulation	Belytschko-Tsay shells; 5 integration points through thickness	
Material model	Temperature and strain rate dependent flow curves combined with anisotropic yield locus model Barlat2000 (Section 3.2); implemented in LS-DYNA via material model MAT_BARLAT_YLD2000	
Failure modeling	Segment-related assignment of single temperature dependent FLCs; implemented in LS-DYNA via MAT_ADD_EROSION	
**Thermal Parameters**		
Contact heat transfer coefficient	2.2 × 10^3^ W/m^2^K [3] for assumed 0.5 N/mm^2^ contact pressure	
Heat transfer due to convection and radiation	0.0–41.5 W/m^2^K (20–500 °C); temperature dependent according to [38]	
Heat transfer to cooling channels	3.0 × 10^3^ W/m^2^K for turbulent flow (water; 20 °C)	
Heat capacity	857–1043 J/kgK (20–450 °C; specimen; AA7075 [39]); 480 J/kgK (steel clamps)	
Thermal conductivity	121–158 W/mK (20–450 °C; specimen; AA7075 [39]); 42 W/mK (steel clamps)	

**Table 4:** Inversely identified parameters of the inductive heat source model.

Parameter	*L*_0_	*L*_1_	*M*	${\dot{q}}_{max}$
Unit	mm	mm	–	W m^−3^
Identified values	4.289	4.919	8.517	1080.9 × 10^6^

## Data Availability

Data is contained within the article.
